# *AmphiTherm*: a comprehensive database of amphibian thermal tolerance and preference

**DOI:** 10.1038/s41597-025-06286-w

**Published:** 2025-11-21

**Authors:** Patrice Pottier, Rachel R. Y. Oh, Pietro Pollo, A. Nayelli Rivera-Villanueva, Yefeng Yang, Sarah Varon, Ana V. Longo, Samantha Burke, Hsien-Yung Lin, José O. Valdebenito, Tatsuya Amano, Szymon M. Drobniak, Shinichi Nakagawa, Natalie Claunch

**Affiliations:** 1 Evolution & Ecology Research Centre, School of Biological, Earth and Environmental Sciences, The University of New South Wales, Sydney, New South Wales Australia; 2 Division of Ecology and Evolution, Research School of Biology, The Australian National University, Canberra, Australian Capital Territory Australia; 3 Department of Biological and Environmental Sciences, University of Gothenburg, Gothenburg, Sweden; 4 German Centre for Integrative Biodiversity Research, Halle-Jena-Leipzig, Leipzig, Germany; 5 Helmholtz Centre for Environmental Research (UFZ), Leipzig, Germany; 6 Centre for Biodiversity and Conservation Science, The University of Queensland, Brisbane, Queensland Australia; 7 School of Environmental and Life Sciences, University of Newcastle, Callaghan, New South Wales Australia; 8 Laboratorio de Biología de la Conservación y Desarrollo Sustentable de la Facultad de Ciencias Biológicas, Universidad Autónoma de Nuevo León, Monterrey, México; 9 School of the Environment, The University of Queensland, Brisbane, Queensland Australia; 10 Department of Biology, University of Florida, Gainesville, Florida USA; 11 Canadian Wildlife Service, Environment and Climate Change Canada, Gatineau, Quebec Canada; 12 Departamento de Zoología, Facultad de Ciencias Naturales y Oceanográficas, Universidad de Concepción, Concepción, Chile; 13 Instituto Milenio Biodiversidad de Ecosistemas Antárticos y Subantárticos (BASE), Santiago, Chile; 14 Institute of Environmental Sciences, Faculty of Biology, Jagiellonian University, Krakow, Poland; 15 Department of Biological Sciences, University of Alberta, Edmonton, Canada; 16 Department of Natural History, Florida Museum of Natural History, Gainesville, Florida USA; 17 USDA APHIS WS National Wildlife Research Center, Florida Field Station, Gainesville, Florida USA

**Keywords:** Herpetology, Animal physiology, Climate-change ecology, Ecophysiology, Macroecology

## Abstract

Thermal traits are crucial to our understanding of the ecology and physiology of ectothermic animals. While rising global temperatures have increasingly pushed research towards the study of upper thermal limits, lower thermal limits and thermal preferences are essential for defining the thermal niche of ectotherms. Through a systematic review of the literature in seven languages, we expanded an existing database of amphibian heat tolerance by adding 1,009 estimates of cold tolerance and 816 estimates of thermal preference across 375 species. *AmphiTherm* is a comprehensive and reproducible database that contains 4,899 thermal trait estimates from a diverse sample of 659 species (~7.5% of all described amphibians) spanning 38 families. Despite its broad geographic coverage, we report evident gaps across amphibian biodiversity hotspots in Africa, most regions of Asia, central South America, and Western Australia. By providing a more holistic understanding of amphibian thermal tolerance and preferences, *AmphiTherm* is a valuable resource for advancing research in evolutionary biology, ecophysiology, and biogeography of amphibians, offering insights that are increasingly needed in changing climates.

## Background & Summary

Thermal trait data are crucial to our understanding of the biology and physiology of ectotherms. The recent increase in broad-scale syntheses of ectotherm thermal physiologies demonstrates recurring interest in how these organisms respond to changing thermal environments^1–10^. Much of this work has focused on traits relating to heat tolerance, reflecting the urgency to predict the impacts of climate warming on natural populations^11–17^. However, climate change also brings an increased probability of extreme weather events, including negative temperature anomalies^18,19^. As such, a sole focus on heat tolerance provides an incomplete picture of ectotherm responses to climate change. A comprehensive understanding of heat tolerance, cold tolerance, and thermal preference is necessary to fully define ectotherm’s thermal niches and predict their responses to climate change. Below, we briefly emphasise the importance of these thermal traits for amphibians (see^8,20,21^ for more in depth discussion).

While the significance of heat tolerance in predicting species’ responses to warming climates is well documented^1^, data on lower thermal limits are equally vital yet often understudied, especially in amphibians, an at-risk, data-deficient group of ectotherms^22^. Lower thermal limits represent the lower boundary of an organism’s thermal niche and have been included in several data syntheses^5,6,8,10^. This trait provides key insights into how species might respond to increasing frequency of extreme cold weather events, which can lead to significant population reduction events known as winterkills^18,19,23^. Gaining understanding of lower thermal limits can thus help predict the sensitivity and resilience of amphibian populations to extreme cold weather events. Moreover, data on lower thermal limits can inform conservation and management strategies, for instance, by identifying microhabitats buffering the effects of extreme cold on activity and survival^24^.

Preferred body temperatures reflect the temperature optimising overall performance and the most favourable microhabitat in the absence of other biotic and abiotic factors^25,26^. Knowledge of preferred body temperatures can thus help predict how climate change will affect species distributions and activity patterns^24,27–29^. In particular, thermal preference data can be used to infer behavioural thermoregulation patterns and the microhabitats available for crucial physiological processes^30–32^. While upper thermal limits can help predict acute survival in the face of extreme heat, gradual warming below the upper thermal limit thresholds can make some areas unsuitable for amphibians’ activity needs^24^. On the contrary, warming can benefit some amphibians in historically cooler climates by increasing activity windows or reducing hibernation times^33^. As such, thermal preference data can help predict the sublethal effects of climate change. Thermal preferences also, for instance, affect susceptibility to pathogens^34–36^, shape the composition of commensal microbes^37,38^, and mediate interactions between host and microbial communities^39^.

Although investigating thermal traits separately provides important knowledge, the study of a combination of thermal traits provides deeper and more comprehensive insights. A simultaneous analysis of upper and lower thermal limits is particularly interesting as it provides an estimation of thermal tolerance breadth^21^—a measure of the thermal envelope ectotherms can occupy in the absence of other abiotic or biotic factors (e.g., competition, resource availability). When thermal preference is integrated with upper and lower thermal limits, the thermal envelope gains shape, providing additional insights to parameterise models and predict activity and survival in changing environments. For instance, leveraging data on thermal limits and preferred temperatures can help infer past, current, and future distributions of ectothermic species, including assessing potential invasion risk^15,40–43^. Parametrising biophysical models with data on thermal limits and preference now also allow more accurate predictions of overall performance, activity windows^17,44^ and microhabitat heterogeneity^17,44,45^, strengthening our ability to predict the impacts of climate change on natural populations^46,47^. From an evolutionary perspective, the integration of different thermal traits can also advance our understanding of the (co)evolution of these traits, and how climate change may shape evolutionary pressures on thermal tolerance and preference^6,29,48–51^.

Thermal trait data can also be used to inform conservation efforts. Comparing thermal niche envelopes among amphibians, their microbiota, and potential pathogens can help predict changes in the microbiome and disease risk^52–57^, providing key information on the environment component of the host-pathogen-environment interactions emphasized with the disease triangle concept^54^. Amphibians are often accessioned into captivity to establish assurance colonies for breeding and eventual reintroduction^58^. Knowledge of the thermal tolerance and preference of a broad range of species from different habitats can help inform the design of enclosures that better simulate natural thermal variability^59–61^. It is well established that temperature influences captive breeding success of amphibians^60^. Amphibian husbandry guides recommend setting thermal environments near the preferred temperature of a species, and when practical, providing thermal gradients and thermal cycling^60,62^. Exposure to environmental conditions via “soft release” or mesocosms prior to reintroduction can also influence survival and success of reintroduction efforts^63,64^. In addition, understanding thermal constraints on activity, demography, and disease risk enhances our ability to identify habitats suitable for repatriation and reintroduction efforts in endangered species^58,65,66^.

Therefore, a holistic understanding of amphibian thermal tolerance limits and preferences is essential for defining the fundamental thermal niche of ectotherms and to project their activity, distribution and survival in rapidly changing environments. Here, we expand an existing database on amphibian upper thermal limits^1^, which has already facilitated global assessments of vulnerability to climate warming^17^. We conducted systematic searches and aggregated lower thermal limits and thermal preference data from the global literature published in seven languages. In doing so, we expanded thermal niche data for 378 species, providing a stronger foundation for research on amphibian ecology, evolution, and conservation.

## Methods

### Reporting

We reported the contributions of each author using the CRediT (Contributor Roles Taxonomy) statement^67^, and MeRIT (Method Reporting with Initials for Transparency) guidelines^68^. We also followed recommendations to maximise the indexing of titles, abstracts, and keywords in databases^69^.

### Literature searches

**Fig. 1:**
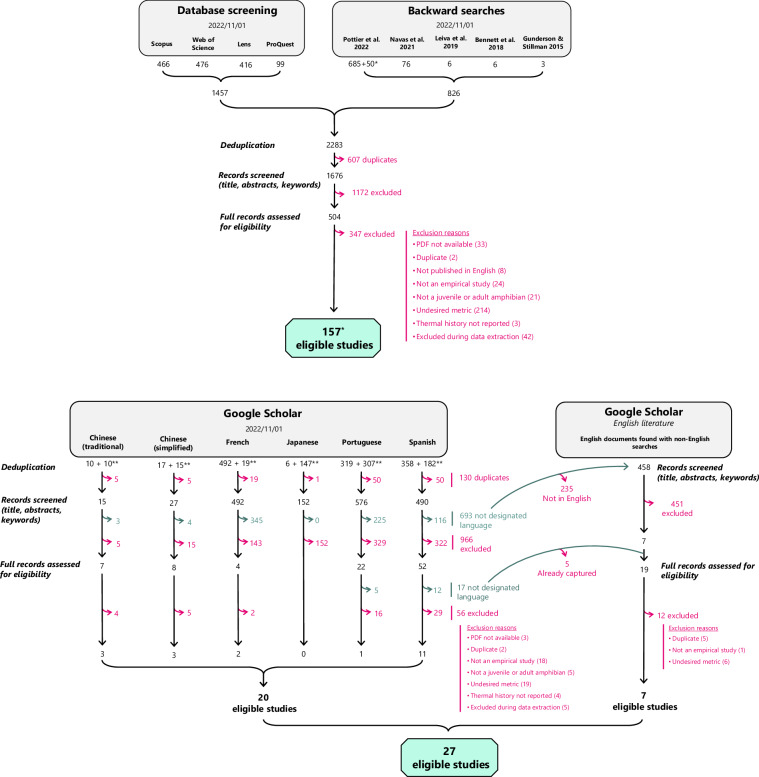
PRISMA Flowchart delineating the databases used to retrieve studies on lower thermal limits and preferred body temperatures, the number of studies obtained at each stage of the screening process, and the reasons for excluding studies. * Two studies published in languages other than English (French, simplified Chinese) were retrieved through English searches. ** Studies from non-English searches done in Google Scholar from^1^. For the workflow used to obtain data on upper thermal limits, see^1^.

We adapted methods from the previous version of the database on amphibian upper thermal limits^1^ to search the literature on thermal physiological traits. We aimed to compile a comprehensive and representative sample of the experimental literature on lower thermal limits and thermal preference in amphibians, complementing the data on upper thermal limits compiled previously (see^1^ for methods specific to upper thermal limits). PPottier accessed Scopus, ISI Web of Science (core collection), Lens, and ProQuest (dissertation & theses) on 01 November 2022 using The University of New South Wales’ institutional subscriptions. Briefly, the search strings were built using as a combination of relevant terms, including “temperature” (and synonyms) and “thermal tolerance or preference” (and synonyms) and “amphibian” (and synonyms) (see Table S1 for details). For studies in English, PPottier modified search strings to accommodate the structure of each database (Table S1) and performed backward searches of previously published reviews of amphibian thermal preference and tolerance^1,4–6,10^. This resulted in a total of 1676 unique documents. We limited the timespan of our searches to 31 May 2021 to match with the timespan represented in the upper thermal limit data^1^. This decision was made to normalise all searches to a single timespan to simplify future database updates. PPottier also performed Traditional and Simplified Chinese, French, Japanese, Portuguese, and Spanish searches in Google Scholar using search strings translated by native speakers (NR, PPottier, PPollo, SN, YY, and RRYO). The searches contained translated singular and plural forms of the following: “amphibian”, “frog”, “toad”, “salamander”, “newt”, “tadpole”, “preferred temperature”, “selected temperature”, “thermal preference”, “Tpref”, “Tsel”, or “CTmin”. Due to search string limitations in Google Scholar (256 characters), each term was assessed in its singular or plural form, and the search producing the largest number of results was selected. We performed searches following the format of (“preferred temperature” OR synonyms) AND (amphibian OR synonyms). We also performed separate searches with “CTmin” and (“Tpref” OR “Tsel”), as these terms are commonly used in the literature to refer to lower thermal limits and preferred temperatures. We (PPottier, NC) opted not to use “thermoregulation” as a synonym for “thermal preference” in our search strategy, as pilot searches revealed that this term often returned studies that did not present experimentally-derived thermal preference values, or studies that were already captured by the other search terms. PPottier used Publish or Perish^70^ to extract bibliographic records from Google Scholar. We also reused studies (designated with ** in Fig. 1) from non-English searches conducted in^1^ as the key terms used successfully retrieved results on cold tolerance. However, we limited the inclusion of studies to those meeting our first two criteria (i.e., studies on amphibians, and published in the targeted language) to reduce the volume of screening. We acknowledge that our searches do not encompass all languages relevant to amphibian thermal physiology research and invite speakers of languages not represented in the current version to contribute to future updates of the database.

### Eligibility criteria

We considered studies that empirically tested lower thermal limits or thermal preference in wild or laboratory amphibians. We included studies on larval, juvenile, metamorphic, and adult amphibians, and distinguished studies that acclimated both embryos and larvae to those acclimating single life stages. We excluded studies solely on embryonic stages due to the lack of comparable methods in embryos—for example, embryos are not capable of movement in a thermal gradient to assess thermal preference, and it is not possible to assess the righting response of embryos. For lower thermal limits, we included studies that measured critical thermal minimum (CTmin)^71^, median lethal temperature (LT50)^72^, or presented data that were convertible to these metrics (e.g., % survival of cohorts tested at different temperatures). We distinguish these metrics in the database. CTmin represents the temperature at which a specific physiological or behavioural endpoint is observed—such as the loss of righting response or a lack of response to prodding—when an organism is exposed to progressively decreasing temperatures (e.g., 1 °C/min). It does not represent the lowest temperature an organism can tolerate, but rather the onset of functional stasis, the point at which the organism is unable to move and incapable of essential survival behaviours such as thermoregulation or predator evasion^71^. This distinction is important, because many ectotherms can recover from temperatures below their CTmin. For instance, some species can recover from freezing to later resume normal function^73^. In contrast, LT50 is the temperature that is lethal to 50% of animals tested and is derived through statistical interpolation from survival rates across a range of temperatures^72^. For thermal preference, we included studies that empirically tested amphibian temperature selection in a thermal gradient or shuttlebox via measures of body temperature (or inferred body temperature from the position in the gradient or shuttlebox). We did not include data reported on amphibian body temperatures from uncontrolled (wild) conditions because available environmental temperatures were not standardised. We only included studies where thermal history (acclimatisation or acclimation temperature) was reported or could be inferred from the dates and coordinates of sampling. PPottier, RRO, PPollo, ANRV, YY, SV, AVL, and NC screened articles for eligibility using Rayyan QCRI^74^. This software facilitated the identification of key terms in titles, abstracts, and keywords to streamline the screening process for large volumes of literature. During data extraction, 47 papers were ultimately excluded for either lacking extractable data or for not complying with our inclusion criteria (43 English, 1 Traditional Chinese, 3 Simplified Chinese). A total of 184 studies were deemed eligible for inclusion in the database. Of these, 157 were identified through formal database searches (comprising one study published in simplified Chinese, and another study in French), while 20 non-English studies and an additional 7 English-language studies were retrieved through Google Scholar (Fig. 1). Therefore, nearly 15% (27/184) of the included studies were retrieved through non-English literature searches. Our literature search methods and screening process is summarised in a PRISMA flowchart^75^ (Fig. 1).

### Data extraction

Data extractions were performed by PPottier (7.7% of estimates extracted), RRYO (6.7%), PPollo (5.3%), ANRV (6.2%), YY (3.0%), AVL (11.0%), SV (20.5%), and NC (45.0%). Note that these values do not add to 100% because some data entries were extracted by two authors. Data were extracted following the protocols described in the previous version of the database^1^. We extracted data directly from text and tables, and primarily used *metaDigitise*^76^ (version 1.0.1) in R^77^ to extract data presented in figures (although note that some authors have used WebPlotDigitizer^78^ (version 4.7)). When data were available in multiple formats (e.g., text and figure), we extracted it from the format with the highest resolution (e.g., data stratified by sex or location rather than aggregated across species). Where possible, we extracted measures of data dispersion (i.e. standard deviation, standard error) to accompany mean estimates. In cases where the raw data was available, we calculated summary statistics (means, standard deviation, sample size) to enhance the accuracy of the analysis. For studies reporting survival rates at different temperatures, we predicted the temperature at which 50% mortality occurred using logistic regression from the *dose.p* function from the MASS package^79^.

We also extracted all additional information presented in the studies to allow investigations of the sources of variation in the data and account for non-independence. We assigned identification numbers to each study, and assigned unique identifiers within each study for each estimate, species, population (individuals of the same species sampled from the same geographical location), and cohort (independent group of individuals within a study). Additional variables included sampling coordinates, acclimation temperatures, ramping rates, life stages, endpoints used to infer cold tolerance, or the duration of exposure to experimental treatments. Additional notes were also taken by each researcher extracting the data to facilitate technical validation. The full list of variables is described in the metadata. Species names were standardised during the extraction to match AmphibiaWeb^80^ and further standardised to match the most comprehensive phylogenetic tree to date^81^ (see *Data Curation*).

### Data curation

To ensure consistency in data extraction across all studies, PPottier and NC extensively reviewed the extracted data to correct typological errors and resolve uncertainties identified during the extraction process. PPottier then curated the data in R^77^ (version 4.4.2), merging the newly extracted data with the previously compiled dataset from^1^. This process involved standardising publication information (publication year, source name) and other variables (e.g., geographical coordinates, IUCN threat status^82^) to ensure uniformity across both datasets. PPottier also standardised species names and taxonomy with phylogenetic information from^81^, which is primarily based on AmphibiaWeb^80^. The combined dataset comprises 324 publications^35,83–405^. Note that 53 of these publications were taken from university dissertations, and some of this work may have now been published e.g.^406,407^.

We also provide a curated version of the database (n = 4,401 estimates), where PPottier excluded data with procedural inconsistencies, incomplete species information (e.g., *Hyloscirtus sp*.), and studies involving animals exposed to toxicants, hormones, high levels of UV radiations, or infected with a pathogen. Procedural inconsistencies included traits measured on a single individual (n = 70 estimates), uncommon or inconsistent methodology (e.g., uncommon endpoint, n = 55), animals starved prior to testing (n = 34), animals that underwent surgery or amputation (n = 16), highly uncertain estimates (n = 14), animals dehydrated prior to testing (n = 14), animals exposed to hypoxic or hypercapnic conditions (n = 13), animals exposed to high levels of UV radiation (n = 5), animals perfused with pH solution (n = 3), estimates without sample sizes or metric of statistical dispersion (n = 3), and animals exposed to predators (n = 2). A script detailing the data curation steps is available at https://github.com/p-pottier/AmphiTherm. This data curation step removed 498 estimates from 15 studies and 45 species. However, we believe that this curated dataset offers broader usability. Nevertheless, we also provide the uncurated version of *AmphiTherm* for users interested in addressing more specific questions (e.g., how toxicants affect thermal tolerance and preference) or identifying existing research gaps within the field.

## Data Records

The *AmphiTherm* database is stored at https://github.com/p-pottier/AmphiTherm, and regularly archived to Zenodo^408^. The repository is organised into four main folders: “data”, “R”, “references”, and “shiny_app”. These contain the metadata (.xlsx), raw, cleaned, and curated data (.csv), code for data curation and for producing the figures (.Rmd), code for deploying the Shiny web application (.R), supplementary data (.csv) and phylogenetic tree (.tre) for producing the figures, and bibliographic files (.ris and.bib) with all the references in the database. The metadata file describes all columns (n = 84) of the *AmphiTherm* database in detail. We have also launched a Shiny web application to help navigate and visualise the database. The Shiny app is available at https://p-pottier.shinyapps.io/AmphiTherm-Explorer/.

Data records are under a CC-BY license, enabling reuse with attribution. Therefore, database users must cite this study as well as the primary data sources to attribute the original authors and comply with copyright regulations.

We aim to conduct updates at regular five-year intervals, following the same systematic methods, to maintain the database as an up-to-date resource for amphibian thermal envelope data. We encourage researchers who possess relevant thermal data not included in the current version to contact us so that the database can be updated to continuously reflect the most comprehensive and current body of knowledge.

## Data Overview

**Fig. 2:**
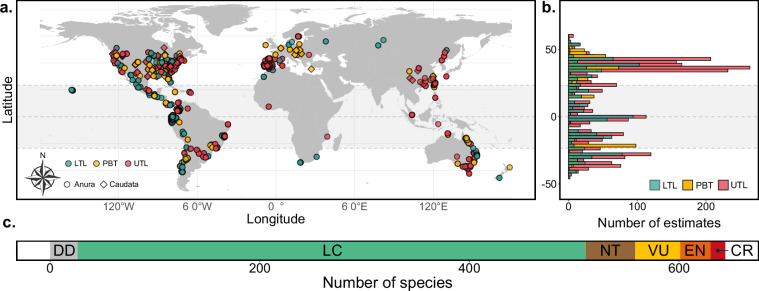
Geographic distribution of thermal tolerance and preference data. (**a**) World map showing the distribution of lower thermal limits (LTL), preferred body temperatures (PBT), and upper thermal limits (UTL) for anurans (circles) and salamanders (triangles). The shaded area represents the tropics. Note that coordinates were unavailable for 775 (15.8%) estimates. (**b**) Latitudinal distribution of estimates for LTL, PBT, and UTL. (**c**) Threat status of species, classified according to the International Union for the Conservation of Nature (IUCN^82^). One species (not displayed) is now extinct.

**Fig. 3:**
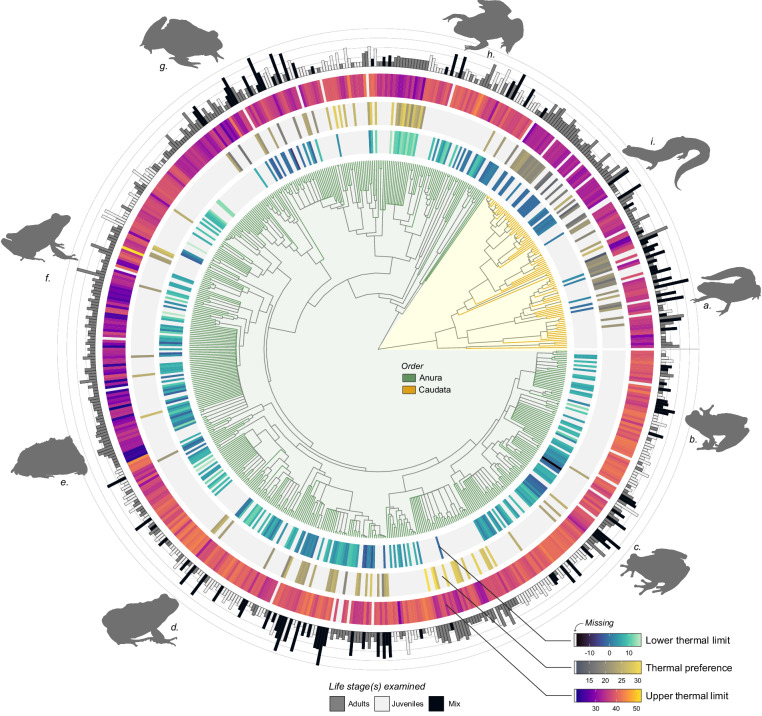
Distribution of mean estimates for three key thermal traits: lower thermal limits (inner heat map), thermal preference (central heat map), and upper thermal limits (outer heat map). The number of estimates for each species is displayed as histograms, scaled on a log2(x + 1) axis for clarity. The histograms are colour-coded according to the life stage assessed in the experiments, with the category “juveniles” comprising larvae, metamorphic, and juvenile stages. Gray colour represents missing data. The phylogeny relationships are based on the consensus tree from^81^. a. *Notophthalmus viridescens*, b. *Dendropsophus ebraccatus*, c. *Hyla cinerea*, d. *Pleurodema thaul*, e. *Ceratophrys cranwelli*, f. *Craugastor longirostris*, g. *Rana pipiens*, h. *Xenopus laevis*, i. *Plethodon cylindraceus*.

**Fig. 4:**
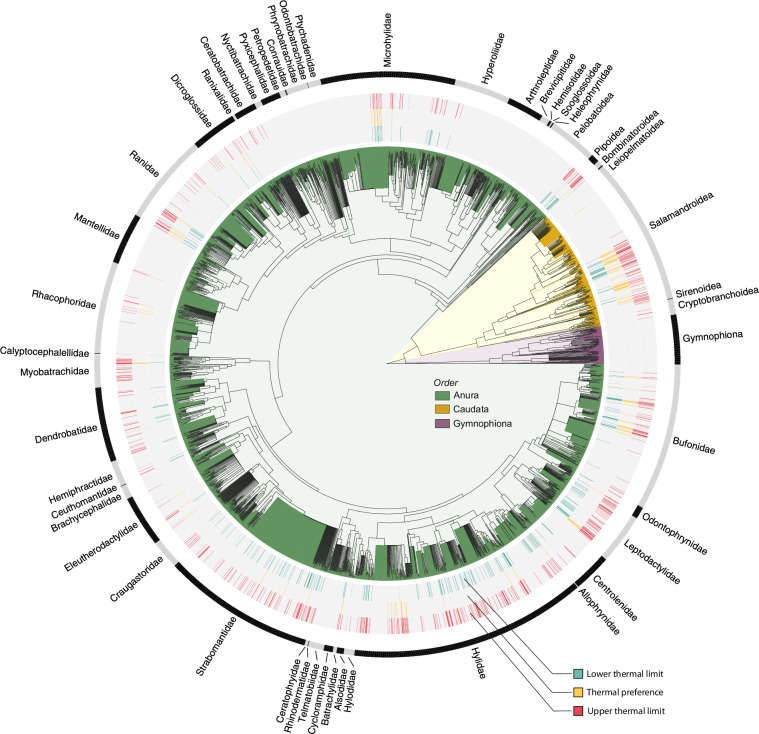
Distribution of thermal trait estimates across the phylogeny of most extant amphibians. Thermal limits and preferences are mapped onto a comprehensive phylogeny of 7,238 species (consensus tree, cf. ^81^) to identify taxonomic biases in existing knowledge. The outer circle presents family names, adapted from^81^.

*AmphiTherm* encompasses 4,899 thermal physiological trait estimates, derived from 324 studies and covering 659 species across 38 families across a broad geographical coverage (Figs. 2, 3). This sample represents ~7.5% of all described amphibian species to date^80^ (Fig. 4). According to the IUCN red list^82^, most species (79.2%) are either not threatened or data-deficient (Fig. 2), yet 47 species are classified as near threatened (NC), 43 as vulnerable (VU), 29 as endangered (EN), 14 as critically endangered (CR), and one species now extinct. Considering that over 40% of amphibians are globally threatened^22^, this suggests that research on amphibian thermal physiology is predominantly conducted on non-threatened species, likely due to the invasive (or lethal) nature of some thermal tolerance experiments and the associated conservation concerns for threatened species. This database contains substantial within-species variation, with an average of 7.43 ± 19.5 (mean ± s.d.) estimates per species, spanning a range of 1 to 292 estimates, with species sampled from an average of 2.51 ± 3.29 populations. Approximately 81% of these estimates include a measure of statistical dispersion (standard deviation, standard error), facilitating their use in weighted (meta-)analyses^409^.

This database update adds thermal data for 375 species, including lower thermal limits for 300 amphibian species and thermal preference data for 137 amphibian species (n = 1,825 estimates; Figs. 3, 4). The majority (98%) of lower thermal limit data are derived from CTmin estimates (990 estimates), with roughly 2% of estimates (19 estimates) derived from lethal limits (LT50). Thermal preference data represent 44% of the database update (816 estimates). This update has a relatively broad phylogenetic coverage, spanning 32 families, with 19.2% of records from salamanders (Figs. 3, 4).

Approximately 62.7% of this database is comprised of upper thermal limit estimates (3074 estimates from 616 species and 212 studies; Figs. 3, 4), highlighting a significant bias towards responses to heat extremes relative to lower thermal limits (1,009 estimates, 300 species, 88 studies) and thermal preferences (816 estimates, 137 species, 114 studies). We found that only 16 studies measured all three thermal traits, covering 59 species (~9% of the species in the dataset; Figs. 3, 4). Upper and lower thermal limits were studied more frequently in tandem (60 studies), allowing to calculate the thermal tolerance breadth (i.e., difference between upper and lower thermal limits) of 276 species (~42% of the species in the dataset; Figs. 3, 4).

Geographically, data were collected on all continents where amphibians occur yet exhibit a strong bias towards Nearctic and European regions (Fig. 2). Large geographic gaps in thermal data are evident across Africa, most regions of Asia, Western Australia, and central South America—regions that are biodiversity hotspots for amphibians (Fig. 2). This is particularly concerning as they constrain our understanding of how species from these underrepresented yet extremely diverse regions^410^ might respond to climate change. We also identified taxonomic gaps in existing sampling where an entire order of amphibians, Gymnophiona, remained unrepresented in the database (Fig. 4). In addition, 1 of 10 families of Caudata and 7 of 36 families of Anura lack thermal limits or preferred body temperature estimates (Fig. 4). This suggests that further efforts are needed to broaden the research scope and better represent the thermal niche of amphibians.

We found that the majority (88.7%) of the literature on amphibian thermal physiological traits was published in English (4,343 estimates). However, non-English language literature contributes a notable and important portion of the knowledge base, accounting for approximately 11.3% of the data. Notably, this includes 289 estimates from publications in traditional Chinese (23 species, 7 studies), 131 estimates from Spanish (40 species, 11 studies), 82 estimates from simplified Chinese (12 species, 10 studies), 28 estimates from Portuguese (10 species, 3 studies), and 26 estimates from French publications (4 species, 3 studies). Including more languages, such as Afrikaans, Arabic, Bengali, Dutch, German, Hindu-Urdu, Korean, Russian, or Swahili in the screening process may help fill some gaps in future updates to the database^411^. Given the historical bias of higher impact publishing outlets against studies on herpetofauna^412^, there are likely a number of studies in non-indexed journals or regional journals in local languages that were not retrieved using our methods.

## Technical Validation

We employed a transparent and reproducible workflow to systematically review over 4,000 studies from five databases and across seven languages. The potential limitations of this database are similar to those described in^1^. First, the methods used for indexing and retrieving studies in Google Scholar are not publicly disclosed^413^, which may undermine reproducibility. However, given the limited coverage of non-English studies in other databases (with 95% and 93% of references in Scopus and Web of Science indexed in English, respectively), Google Scholar remains one of the most suitable tools to synthesise across multiple non-English languages at present^414,415^. Second, different authors extracted data from the original studies, introducing the possibility of individual errors. To ensure consistency and accuracy, all extracted data were subsequently cross-checked and standardised by NC and PPottier (see *Data Curation*) to minimise the risk of bias and strengthen the reliability of the dataset.

## Usage Notes

We anticipate that this database will facilitate a wide range of novel analyses and investigations that may be difficult to foresee at this time, but we are excited to see how these data advance research in amphibian biology and conservation. Our recommendations for using this resource are straightforward: we encourage researchers to have strong foundations in thermal ecology and amphibian biology, and to carefully consider the best approaches for integrating these data into their own investigations.

The database represents a comprehensive compilation of studies employing diverse approaches and experimental designs. Given that we cannot anticipate the full scope of research applications, we have made the entire dataset available to allow users to filter and customise the data as needed. We strongly encourage users to clearly document their analytical steps to ensure reproducibility. However, we emphasise that this database version includes data from animals tested under atypical conditions (e.g., amputations, chemical exposure), or from experiments without replication (e.g. data from a single individual). To accommodate most research needs, we therefore also provide a curated version of the database where we excluded data with procedural inconsistencies, incomplete species-level information, and data involving animals exposed to toxicants, hormones, excessive UV radiation, or pathogens. This curated version of the database is likely more suited for research in ecophysiology, though users with more specialised research questions may find value in the complete dataset. Scripts detailing the data cleaning and curation processes are available at https://github.com/p-pottier/AmphiTherm and should provide further guidance for researchers in tailoring the dataset to their specific research needs. We also provide a Shiny web application (https://p-pottier.shinyapps.io/AmphiTherm-Explorer/) to facilitate data exploration and filtering.

As described in the first iteration of this database^1^, the data contain inherent sources of non-independence as multiple estimates were extracted from each study, species, population (multiple sampling locations from each species), and cohort (e.g., repeated measures on the same individuals). We recommend that users use phylogenetically-informed statistical models with hierarchical random-effect structures to account for and partition sources of variation^408,416^. Users should also account for variations in sampling effort (sample size differences), for instance, by weighting estimates by the inverse of their sampling variance^408^. Employing hierarchical models that incorporate sampling variance can help address issues of biological and methodological non-independence, enabling more accurate inferences of the factors driving variation in the data^408^. Most (81%) estimates are associated with a measure of dispersion (standard deviation or standard error), species information is standardised to published phylogenetic information^81^, and unique identifiers have been assigned to each study, species, population, and cohort. These features make *AmphiTherm* readily applicable for use in complex statistical models aimed at uncovering the drivers of thermal tolerance and preferences in amphibians.

As described in previous studies, thermal traits in amphibians are influenced by multiple variables, including acclimation temperature, acclimation time, ramping rate, endpoint metrics, body size, sex, assay duration, and geographic origin, among others^8,20,21^. We recommend careful attention to these variables, with consideration of incorporating sources of methodological or biological variation as covariates in statistical models, to better capture the complexities of amphibian thermal ecology. Finally, it is important to note that due to the data gaps in hotspots of amphibian diversity, the data herein represent only a subset of total amphibian diversity, and subsequent analyses should acknowledge this limitation. Formally assessing the extent to which geographic and taxonomic biases may influence ecological inferences is an important avenue for research.

## Supplementary information

Supplementary Information

## Data Availability

All data and materials needed to reproduce this study are available at https://github.com/p-pottier/AmphiTherm, which is archived permanently in Zenodo^408^.

## Code availability

The code used to process the data and produce the figures for this manuscript is available at https://github.com/p-pottier/AmphiTherm, which is archived permanently in Zenodo^408^.
