# Supplementary material for: AmphiTherm: a comprehensive database of amphibian thermal tolerance and preference
Source: Sci Data. 2025 Nov 21;12:1987. doi: 10.1038/s41597-025-06286-w (PMC12727874; doi:10.1038/s41597-025-06286-w)
Supplement: Supplementary file 1 — Supplementary Information [file 41597_2025_6286_MOESM1_ESM.docx]

**Supplementary Information**

**Table S1: Search strings used for the different databases.**

| **Database** | **Search strings** |
| --- | --- |
| Scopus | TITLE-ABS-KEY("temperature*" OR "thermal" OR "cold*" OR "cool*") AND TITLE-ABS-KEY("cold tolerance*" OR "tolerance* to cold" OR "thermal min*" OR "CTmin" OR "CT min" OR "chill coma" OR "cold stress tolerance*" OR "tolerance to cold stress" OR "cold stupor" OR "cold resistance" OR "resistance to cold stress" OR "supercooling point" OR "SCP" OR "crystal* temperature*" OR "cold hardiness" OR "freez* tolerance" OR "tolerance to freezing" OR "preferred temperature*" OR "preferred body temperature*" OR "temperature preference*" OR "selected temperature*" OR "selected body temperature*" OR "thermal prefer*" OR "temperature* prefer*" OR "temperature* select*" OR "thermal selection") AND TITLE-ABS-KEY("amphibia*" OR "frog*" OR "toad*" OR "salamand*" OR "newt" OR "newts" OR "tadpole*" OR "metamorph" OR "metamorphs" OR "caecili*" OR "rhinatrema*" OR "ichthyophi*" OR "scolecomorph*" OR "chikil*" OR "herpelidae" OR "typhlonect*" OR "indotyphlid*" OR "dermophi*" OR "siphonop*" OR "caudata" OR "urodela" OR "cryptobranch*" OR "hynobiid*" OR "sirenidae" OR "ambystoma*" OR "dicamptodon*" OR "proteidae" OR "rhyacotriton*" OR "amphium*" OR "plethodon*" OR "anura*" OR "ascaph*" OR "leiopelma*" OR "bombina*" OR "alyt*" OR "rhinophryn*" OR "pipidae" OR "xenopus" OR "scaphiop*" OR "pelodyt*" OR "megophry*" OR "pelobat*" OR "heleophryn*" OR "calyptocephalell*" OR "myobatrach*" OR "rhinoderma*" OR "alsod*" OR "hylod*" OR "batrachyl*" OR "cycloramph*" OR "telmatob*" OR "ceratophry*" OR "hemiphract*" OR "hyla*" OR "hylidae" OR "bufo*" OR "leptodactyl*" OR "odontophryn*" OR "allophryn*" OR "centrolen*" OR "dendrobat*" OR "ceuthomanti*" OR "eleutherodactyl*" OR "brachycephalidae" OR "craugastor*" OR "strabomantidae" OR "pristimantis" OR "nasikabatrach*" OR "soogloss*" OR "microhyl*" OR "arthroleptid*" OR "hyperol*" OR "brevicipitidae" OR "hemisus" OR "odontobatrach*" OR "phrynobatrach*" OR "ptychaden*" OR "conraua" OR "petropedet*" OR "pyxicephal*" OR "micrixalus" OR "nyctibatrach*" OR "ranixalidae" OR "ceratobatrach*" OR "dicroglossidae" OR "rana" OR "ranidae" OR "rhacophor*" OR "mantellidae") AND (EXCLUDE(PUBYEAR , 2022)) |
| Web of Science (core collection) | TS=("temperature*" OR "thermal" OR "cold*" OR "cool*") AND TS=("cold tolerance*" OR "tolerance* to cold" OR "thermal min*" OR "CTmin" OR "CT min" OR "chill coma" OR "cold stress tolerance*" OR "tolerance to cold stress" OR "cold stupor" OR "cold resistance" OR "resistance to cold stress" OR "supercooling point" OR "SCP" OR "crystal* temperature*" OR "cold hardiness" OR "freez* tolerance" OR "tolerance to freezing" OR "preferred temperature*" OR "preferred body temperature*" OR "temperature preference*" OR "selected temperature*" OR "selected body temperature*" OR "thermal prefer*" OR "temperature* prefer*" OR "temperature* select*" OR "thermal selection") AND TS=("amphibia*" OR "frog*" OR "toad*" OR "salamand*" OR "newt" OR "newts" OR "tadpole*" OR "metamorph" OR "metamorphs" OR "caecili*" OR "rhinatrema*" OR "ichthyophi*" OR "scolecomorph*" OR "chikil*" OR "herpelidae" OR "typhlonect*" OR "indotyphlid*" OR "dermophi*" OR "siphonop*" OR "caudata" OR "urodela" OR "cryptobranch*" OR "hynobiid*" OR "sirenidae" OR "ambystoma*" OR "dicamptodon*" OR "proteidae" OR "rhyacotriton*" OR "amphium*" OR "plethodon*" OR "anura*" OR "ascaph*" OR "leiopelma*" OR "bombina*" OR "alyt*" OR "rhinophryn*" OR "pipidae" OR "xenopus" OR "scaphiop*" OR "pelodyt*" OR "megophry*" OR "pelobat*" OR "heleophryn*" OR "calyptocephalell*" OR "myobatrach*" OR "rhinoderma*" OR "alsod*" OR "hylod*" OR "batrachyl*" OR "cycloramph*" OR "telmatob*" OR "ceratophry*" OR "hemiphract*" OR "hyla*" OR "hylidae" OR "bufo*" OR "leptodactyl*" OR "odontophryn*" OR "allophryn*" OR "centrolen*" OR "dendrobat*" OR "ceuthomanti*" OR "eleutherodactyl*" OR "brachycephalidae" OR "craugastor*" OR "strabomantidae" OR "pristimantis" OR "nasikabatrach*" OR "soogloss*" OR "microhyl*" OR "arthroleptid*" OR "hyperol*" OR "brevicipitidae" OR "hemisus" OR "odontobatrach*" OR "phrynobatrach*" OR "ptychaden*" OR "conraua" OR "petropedet*" OR "pyxicephal*" OR "micrixalus" OR "nyctibatrach*" OR "ranixalidae" OR "ceratobatrach*" OR "dicroglossidae" OR "rana" OR "ranidae" OR "rhacophor*" OR "mantellidae") NOT PY=(2022) |
| Lens | ("temperature*" OR "thermal" OR "cold*" OR "cool*") AND ("cold tolerance*" OR "tolerance* to cold" OR "thermal min*" OR "CTmin" OR "CT min" OR "chill coma" OR "cold stress tolerance*" OR "tolerance to cold stress" OR "cold stupor" OR "cold resistance" OR "resistance to cold stress" OR "supercooling point" OR "SCP" OR "crystal* temperature*" OR "cold hardiness" OR "freez* tolerance" OR "tolerance to freezing" OR "preferred temperature*" OR "preferred body temperature*" OR "temperature preference*" OR "selected temperature*" OR "selected body temperature*" OR "thermal prefer*" OR "temperature* prefer*" OR "temperature* select*" OR "thermal selection") AND ("amphibia*" OR "frog*" OR "toad*" OR "salamand*" OR "newt" OR "newts" OR "tadpole*" OR "metamorph" OR "metamorphs" OR "caecili*" OR "rhinatrema*" OR "ichthyophi*" OR "scolecomorph*" OR "chikil*" OR "herpelidae" OR "typhlonect*" OR "indotyphlid*" OR "dermophi*" OR "siphonop*" OR "caudata" OR "urodela" OR "cryptobranch*" OR "hynobiid*" OR "sirenidae" OR "ambystoma*" OR "dicamptodon*" OR "proteidae" OR "rhyacotriton*" OR "amphium*" OR "plethodon*" OR "anura*" OR "ascaph*" OR "leiopelma*" OR "bombina*" OR "alyt*" OR "rhinophryn*" OR "pipidae" OR "xenopus" OR "scaphiop*" OR "pelodyt*" OR "megophry*" OR "pelobat*" OR "heleophryn*" OR "calyptocephalell*" OR "myobatrach*" OR "rhinoderma*" OR "alsod*" OR "hylod*" OR "batrachyl*" OR "cycloramph*" OR "telmatob*" OR "ceratophry*" OR "hemiphract*" OR "hyla*" OR "hylidae" OR "bufo*" OR "leptodactyl*" OR "odontophryn*" OR "allophryn*" OR "centrolen*" OR "dendrobat*" OR "ceuthomanti*" OR "eleutherodactyl*" OR "brachycephalidae" OR "craugastor*" OR "strabomantidae" OR "pristimantis" OR "nasikabatrach*" OR "soogloss*" OR "microhyl*" OR "arthroleptid*" OR "hyperol*" OR "brevicipitidae" OR "hemisus" OR "odontobatrach*" OR "phrynobatrach*" OR "ptychaden*" OR "conraua" OR "petropedet*" OR "pyxicephal*" OR "micrixalus" OR "nyctibatrach*" OR "ranixalidae" OR "ceratobatrach*" OR "dicroglossidae" OR "rana" OR "ranidae" OR "rhacophor*" OR "mantellidae")  Year Published = (1900 - 2021)  Field of Study = (excl Botany , excl Physics , excl Geology , excl Materials science , excl Context (language use) , excl Geochemistry , excl Internal medicine , excl Mutant , excl Arabidopsis , excl Condensed matter physics , excl Computer science , excl Biophysics , excl Biotechnology , excl Computational biology , excl Horticulture , excl Aquaporin , excl Crop , excl Mineralogy , excl Nanotechnology , excl Arabidopsis thaliana , excl Chemical physics) |
| Proquest (Dissertation and Theses) | (noft(cold tolerance*) OR noft(CTmin*) OR noft(preferred temperature*) OR noft(selected temperature*)) AND (noft(amphibia*) OR noft(frog*) OR noft(toad*) OR noft(anura*) OR noft(tadpole*) OR noft(salamand*) OR noft(newts)) |
| Google Scholar (French) | (”température préférée” OR “température sélectionnée” OR “température choisie” OR “préférences thermiques”) AND (amphibiens OR grenouille OR crapaud OR salamandres OR triton OR têtards OR Amphibia OR Caudata OR Anura OR batracien OR anoure)  CTmin AND (“amphibiens” OR grenouille OR crapaud OR “salamandres” OR triton OR têtards OR batracien OR anoure)   (Tpref OR Tsel) AND (“amphibiens” OR grenouille OR crapaud OR “salamandres” OR triton OR têtards OR batracien OR anoure) |
| Google Scholar (Japanese) | (好適温度 OR 選択温度 OR 温度嗜好性) AND (両生類 OR カエル OR ヒキガエル OR サンショウウオ OR イモリ OR おたまじゃくしOR “Amphibia” OR “Caudata” OR “Anura”)   CTmin AND (両生類 OR カエル OR ヒキガエル OR サンショウウオ OR イモリ OR オタマジャクシ)  (Tpref OR Tsel) AND (両生類 OR カエル OR ヒキガエル OR サンショウウオ OR イモリ OR オタマジャクシ) |
| Google Scholar (Portuguese) | (“temperatura preferida” OR “temperatura selecionada” OR “preferência termal”) AND (anfíbio OR “rã” OR sapos OR salamandra OR tritão OR girino OR Amphibia OR Caudata OR Anura OR anuros)   CTmin AND (anfíbio OR “rã” OR sapos OR salamandra OR tritão OR girino)  (Tpref OR Tsel) AND (anfíbio OR “rã” OR sapos OR salamandra OR tritão OR girino) |
| Google Scholar (simplified Chinese) | (合适温度 OR 选温度 OR 耐热程度) AND (两栖动物 OR 青蛙 OR 蛤蟆 OR 蝾螈 OR 蝌蚪 OR 小鲵 OR 大鲵 OR Amphibia OR Caudata OR Anura)   CTmin AND (两栖动物 OR 青蛙 OR 蛤蟆 OR 蝾螈 OR 蝌蚪 OR 小鲵 OR 大鲵)  (Tpref OR Tsel) AND (两栖动物 OR 青蛙 OR 蛤蟆 OR 蝾螈 OR 蝌蚪 OR 小鲵 OR 大鲵) |
| Google Scholar (traditional Chinese) | (偏好溫度 OR 溫度選擇OR 熱偏好) AND (兩棲類 OR 蛙青蛙 OR 蟾蟾蜍癩蝦蟆 OR 螈蠑螈OR 蝌蚪 OR 鯢小鯢山椒魚 OR 鯢大鯢娃娃魚OR Amphibia OR Caudata OR Anura)  CTmin AND (兩棲類 OR 蛙青蛙 OR 蟾蟾蜍癩蝦蟆 OR 螈蠑螈OR 蝌蚪 OR 鯢小鯢山椒魚 OR 鯢大鯢娃娃魚)   (Tpref OR Tsel) AND (兩棲類 OR 蛙青蛙 OR 蟾蟾蜍癩蝦蟆 OR 螈蠑螈OR 蝌蚪 OR 鯢小鯢山椒魚 OR 鯢大鯢娃娃魚) |
| Google Scholar (Spanish) | (“temperatura preferida” OR “temperatura seleccionada” OR “preferencias térmicas”) AND (anfibio OR rana OR sapo OR salamandra OR tritón OR renacuajo OR Amphibia OR Caudata OR Anura OR anuros)   CTmin AND (anfibio OR rana OR sapo OR salamandra OR tritón OR renacuajo or Anuros)  (Tpref OR Tsel) AND (anfibio OR rana OR sapo OR salamandra OR tritón OR renacuajo or Anuros |
